# Evaluation of Neonatal Screening Programs for Tyrosinemia Type 1 Worldwide

**DOI:** 10.3390/ijns10040082

**Published:** 2024-12-16

**Authors:** Allysa M. Kuypers, Marelle J. Bouva, J. Gerard Loeber, Anita Boelen, Eugenie Dekkers, Konstantinos Petritis, C. Austin Pickens, The ISNS Representatives, Francjan J. van Spronsen, M. Rebecca Heiner-Fokkema

**Affiliations:** 1 Section of Metabolic Diseases, Beatrix Children’s Hospital, University Medical Center Groningen, University of Groningen, 9700 RB Groningen, The Netherlands; a.m.dijkstra@umcg.nl (A.M.K.); f.j.van.spronsen@umcg.nl (F.J.v.S.); 2 Centre for Health Protection, National Institute for Public Health and the Environment (RIVM), 3720 BA Bilthoven, The Netherlands; marelle.bouva@rivm.nl; 3 International Society for Neonatal Screening (ISNS) Office, 3721 CK Bilthoven, The Netherlands; office-manager@isns-neoscreening.org; 4 Endocrine Laboratory, Department of Clinical Chemistry, Amsterdam Gastroenterology, Endocrinology & Metabolism, Amsterdam UMC, University of Amsterdam, 1105 AZ Amsterdam, The Netherlands; a.boelen@amsterdamumc.nl; 5 Centre for Population Research, National Institute for Public Health and the Environment (RIVM), 3720 BA Bilthoven, The Netherlands; eugenie.dekkers@rivm.nl; 6 Newborn Screening and Molecular Biology Branch, Division of Laboratory Sciences, National Center for Environmental Health, Centers for Disease Control and Prevention, Atlanta, GA 30341, USA; nmo3@cdc.gov (K.P.); ogh6@cdc.gov (C.A.P.); 7 Laboratory of Metabolic Diseases, Department of Laboratory Medicine, University Medical Center Groningen, University of Groningen, P.O. Box 30 001, 9700 RB Groningen, The Netherlands

**Keywords:** tyrosinemia type 1, neonatal screening, dried blood spots, inborn metabolic disease, succinylacetone, tyrosine

## Abstract

In The Netherlands, newborn screening (NBS) for tyrosinemia type 1 (TT1) uses dried blood spot (DBS) succinylacetone (SUAC) as a biomarker. However, high false-positive (FP) rates and a false-negative (FN) case show that the Dutch TT1 NBS protocol is suboptimal. In search of optimization options, we evaluated the protocols used by other NBS programs and their performance. We distributed an online survey to NBS program representatives worldwide (*N* = 41). Questions focused on the organization and performance of the programs and on changes since implementation. Thirty-three representatives completed the survey. TT1 incidence ranged from 1/13,636 to 1/750,000. Most NBS samples are taken between 36 and 72 h after birth. Most used biomarkers were DBS SUAC (78.9%), DBS Tyrosine (Tyr; 5.3%), or DBS Tyr with second tier SUAC (15.8%). The pooled median cut-off for SUAC was 1.50 µmol/L (range 0.3–7.0 µmol/L). The median cut-off from programs using laboratory-developed tests was significantly higher (2.63 µmol/L) than the medians from programs using commercial kits (range 1.0–1.7 µmol/L). The pooled median cut-off for Tyr was 216 µmol/L (range 120–600 µmol/L). Overall positive predictive values were 27.3% for SUAC, 1.2% for Tyr solely, and 90.1% for Tyr + SUAC. One FN result was reported for TT1 NBS using SUAC, while three FN results were reported for TT1 NBS using Tyr. The NBS programs for TT1 vary worldwide in terms of analytical methods, biochemical markers, and cut-off values. There is room for improvement through method standardization, cut-off adaptation, and integration of new biomarkers. Further enhancement is likely to be achieved by the application of post-analytical tools.

## 1. Introduction

Tyrosinemia type 1 (TT1, OMIM#276700) is a rare inborn metabolic disease, caused by a deficiency of the fumarylacetoacetate hydrolase (FAH) enzyme, which disrupts the catabolic pathway of tyrosine (Tyr). Consequently, toxic metabolites, including fumarylacetoacetate and succinylacetone (SUAC), accumulate, resulting in liver failure, hepatic malignancy, mainly hepatocellular carcinoma (HCC) and hepatoblastoma, renal tubular dysfunction, and porphyria crises with neuropathy [1,2]. Without liver transplantation and in the absence of other available treatment, TT1 used to be lethal early in childhood [1,2]. Fortunately, the outcome for TT1 patients significantly improved with the introduction of 2-(2-nitro-4-trifluoromethylbenzoyl)-1,3-cyclohexanedione (NTBC or Nitisinone^®^), which prevents accumulation of toxic metabolites by blockage of Tyr degradation some steps proximal to the FAH defect [3]. 

The severity of the disease and the gained benefits from early diagnosis and treatment with NTBC make TT1 a good candidate for neonatal screening (NBS) [2,4,5]. And although TT1 was not one of the core diseases in the early NBS programs, with the introduction of mass spectrometry in NBS, it is increasingly added to the extended NBS programs worldwide [6,7,8]. In the Netherlands, NBS for TT1 started in 2007, using dried blood spot (DBS) Tyr as informative marker with a cut-off of 500 µmol/L but was ceased soon after because of high numbers of false-positive (FP) screening results [9]. In late 2008, TT1 screening was reintroduced using DBS SUAC as informative marker with a cut-off of 1.50 µmol/L [10,11]. Quality improvements and the introduction of new assays and mass spectrometers in the Dutch screening laboratories led to multiple changes in the cut-off for SA over the years, presently resulting in a cut-off value of 0.60 μmol/L.

Although DBS SUAC is said to have high positive and negative predictive values for TT1 [4,12], it is associated with analytical challenges, resulting in poor recovery of SUAC and falsely high concentrations in case of contamination of the mass spectrometers [13]. Moreover, since the start of SUAC NBS for TT1 in the Netherlands, 57% of positive screening results have proven to be FP results, and in 2020, a false-negative (FN) TT1 patient emerged [14,15]. This clearly shows that the Dutch NBS program for TT1 is suboptimal, especially since the predictive values of SUAC appear to be much higher in other countries [12].

In search of optimization options for the TT1 NBS in the Netherlands, we first tried to perform a review study, summarizing the protocols and performances of TT1 NBS programs. However, we soon abandoned this plan, as we only found a very limited number of publications on ongoing official screening programs for TT1. Therefore, in this study, we evaluated the protocols used by other NBS programs and their performance in an international survey. By this, we aim to summarize differences in, among other things, biochemical markers, cut-offs, and FP and FN results between TT1 screening programs worldwide. 

## 2. Materials and Methods

### 2.1. Participants

We performed a web-based survey study on TT1 screening worldwide, targeting the representatives of the NBS programs of the countries or regions screening for TT1. In a collaboration between the Groningen University Medical Center (UMCG; F.J.v.S., R.H.), the endocrine laboratory of the Amsterdam Medical Center (A.B.), the Dutch National Institute for Public Health and the Environment (RIVM; M.J.B., E.D.), and the International Society of Neonatal Screening (ISNS; J.G.L.), we created a list of possible representatives (from the ISNS database), who were asked to participate in the survey.

### 2.2. Survey Creation and Content

The survey was created, and results from the survey were recorded using the web-based survey platform Qualtrics (https://www.qualtrics.com/). The first draft of the survey was created by A.M.K. and checked by F.J.v.S. and R.H. Thereafter, J.G.L. (ISNS) and M.J.B. and E.D. (RIVM) gave their feedback on this version, resulting in the final version of the survey. Finally, a list of 16 questions was produced (see File S1). The survey was available in English. Questions focused on the organization and performance of the program and on changes since the implementation of the program. Answers to the questions were either open or closed. 

### 2.3. Survey Distribution

Forty-one (41) representatives of the NBS programs in countries/regions that are currently known to have screening for TT1 installed received an initial email to invite them to participate in the survey (ISNS mailing list). Moreover, an email was sent to a ListServe of people in the US that are connected to NBS. The email contained a short background and study design, an invitation for participation, instructions for completing the survey, privacy regulations, and a link to the survey. On top, it contained an invitation to the recipient to forward the email if they felt another person would be more suited to complete this survey. 

A reminder email was sent 2 weeks after the initial email, urging possible participants to fill in the survey. Aiming to improve participation, a third and fourth reminder was sent to non-responders at one and two months after the initial email. Another personal reminder was sent to those that started but did not complete the survey, or in case clarification of the answers was needed. Unfortunately, despite our efforts, some key programs did not respond. To be able to include some information from these region’s NBS programs, we looked at other ways of data collection. For Germany, data were supplemented from the German national screening (DGNS) reports [16]. For the United States (US), anonymized data from NBS performance were provided by the Centers for Disease Control and Prevention (CDC). As part of their Newborn Screening Quality Assurance Program (NSQAP), the CDC periodically evaluates the affiliated laboratories’ analytical performance for specific assays, such as SUAC. This is achieved by providing DBS specimens, certified for homogeneity, accuracy, stability, and suitability for assays from different commercial sources and laboratory-developed tests, and gathering data on testing performance, including, among other things, measured values and cut-offs [17]. All CDC NSQAP data were blinded and aggregated before analysis, so no individual NSQAP participant data were provided. Data on laboratory SUAC measurements worldwide gathered from these programs were used in this study. Results from the survey were written using the CHERRIES checklist (see File S2) for proper reporting of results from web-based surveys).

### 2.4. Statistical Analysis

Only complete surveys were analyzed. Survey and blinded CDC data were analyzed using descriptive statistics and simple regression analyses. From the data on the number of neonates screened and the number of TPs and FPs, we calculated the incidence of TT1, the FNs, the positive predictive value (PPV), and the negative predictive value (NPV). Multiple responses for the same country or region were checked for complete similarity and reported as one result. In case of discrepancies, the participants were contacted for clarification of the answers. 

### 2.5. Ethical Statement

This study did not include clinical research with individual human subjects as meant by the Dutch Medical Research Act involving human subjects. Therefore, asking for medical ethical approval was deemed unnecessary. 

## 3. Results

### 3.1. Responses

**Figure 1:**
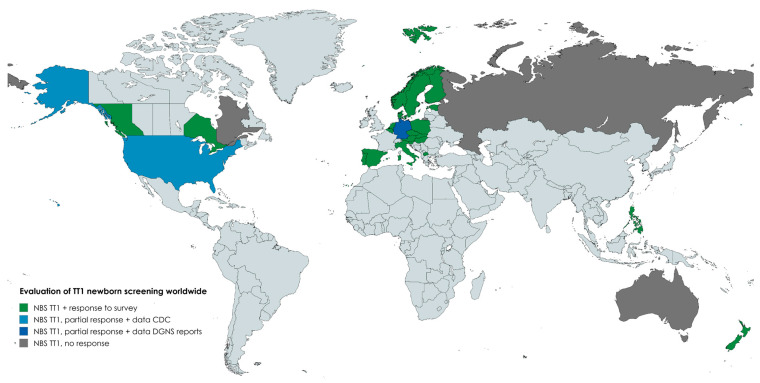
An overview of countries and regions implementing NBS for TT1 worldwide. Overview of countries and regions implementing NBS for TT1 worldwide and whether or not they responded to our survey.

From the 41 surveys sent to recipients from the established ISNS mailing list, data were obtained from 32 responders, yielding a response rate of 78%. We received no response from Australia, Quebec (Canada), or Russia, and from 6/11 regional screening labs from Germany. From the US ListServe, we only received three responses, of which only one (Kansas) completed (no response rate could be calculated from this ListServe as the mailing list contained multiple contacts working for the same NBS program). As far as possible, the information from the missing German screening labs was supplemented by data from the DGNS reports [16]. Combined, we received a total of 33 completed surveys. In the lack of further responses from the US, we supplemented the rest of the laboratory data with the help of the CDC. A complete overview of the responding countries can be seen in Figure 1. 

### 3.2. Screening Practices Worldwide

Table 1 shows the complete overview of all demographic information from the NBS programs for TT1 worldwide gathered from our survey. In addition, from the total number screened, the number of true positives, FP, and FN, the incidence of TT1, as well as the PPV and the NPV of the different screening programs, were calculated. The most important results are summarized below. 

#### 3.2.1. Time of Sampling

In most NBS programs, dried blood samples are taken somewhere between 24 and 96 h after birth, with most being sampled between 36 and 72 h. However, many differences were seen, with programs sampling before 24 h after birth (Kansas, USA) until even up to two years (Alberta, Canada).

#### 3.2.2. Biochemical Screening Marker

In addition to the 33 responses, used screening markers from five additional screening labs were retrieved from the German DGSN reports, giving a total of 38 results for this part. Most countries (30/38 = 78.9%) currently use SUAC as the primary biochemical marker for TT1, with three countries using Tyr as a second tier in addition to SUAC (Ontario, Canada, and Denmark, and Estonia, 3/30 = 10%), two countries using SUAC/Tyr, SUAC/Phe, and SUAC/Met ratios as a second tier next to SUAC (Slovenia and Galicia, Spain, 2/30 = 6.7%), and one country using FAH sequencing as a second tier next to SUAC (Norway, 1/30 = 3.3%) (Table 1). Six countries/regions (Belgium, Flanders/Belgium, and Wallonia/Hungary/Poland/Portugal/Singapore, 15.8%) screen for TT1 using Tyr as primary biomarker but with SUAC as second tier. Two countries (North Macedonia and Slovakia, 5.3%) screen for TT1 solely using Tyr as a biomarker. 

#### 3.2.3. Cut-Offs 

**Figure 2:**
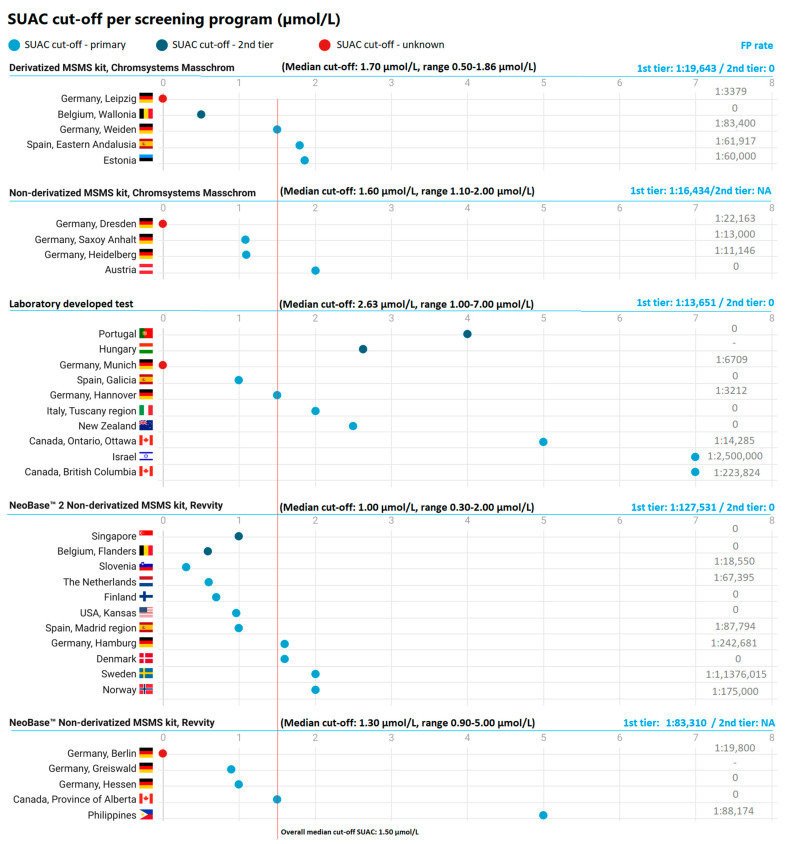
An overview of SUAC cut-off values across screening programs (umol/L). Each dot represents the SUAC cut-off (µmol/L, X-axes) per screening program (Y-axes), grouped per analytical assay. FP-rates are based on FP incidence (number of FP cases found per total screened). For the combined FP-rate per analytical method, the number of FP results and total number screened of all countries using the same analytical method are combined.

*SUAC*: From the 36 countries/regions screening with SUAC (either primary or as a second tier), 31 cut-offs for SA were collected (four cut-offs could not be retrieved from the DGSN reports, and one country screening with SUAC as second tier only disclosed their cut-off for Tyr). A median was calculated from all SUAC cut-offs, which was 1.50 µmol/L (range of 0.3–7.0 µmol/L). Sixteen out of 31 programs with available SUAC cut-offs (51.6%) screen with a value on or below the pooled median, and 15 programs (48.4%) screen with a cut-off above the overall median (Figure 2).

**Figure 3:**
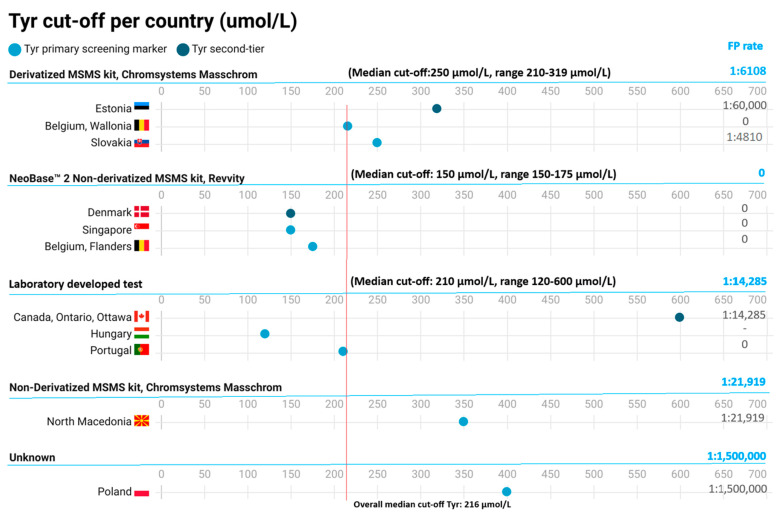
An overview of Tyr cut-off values across screening programs (umol/L). Each dot represents the cut-off of Tyr (µmol/L) per screening program, grouped per analytical assay. FP-rates are based on FP incidence (number of FP cases found per total screened). For the combined FP-rate per analytical method, the number of FP results and total number screened of all countries using the same analytical method are combined.

*TYR*: Eleven programs screen with Tyr (eight as primary marker, and three as second tier to SUAC in a two-tier protocol). The median cut-off value for Tyr was 216 µmol/L (range of 120–600 µmol/L). Six out of eleven programs (54.5%) screen with a cut-off below or similar to the median, and five programs (45.5%) screen with a cut-off above the median (Figure 3).

#### 3.2.4. Analytical Assays and Filter Paper

From the 38 programs, 23 (60.5%) use the Whatman 903 collection filter paper, and 15 screening programs (39.5%) use the Revvity (formerly PerkinElmer) 226 collection paper. There were five main analytical assays that are used among the different screening programs, namely: (1) the non-derivatized MSMS kit from Chromsystems, Masschrom, used by six screening programs (15.7%); (2) the derivatized MSMS kit from Chromsystems, Masschrom, used by five screening programs (13.2%); (3) the NeoBase™ non-derivatized MSMS kit from Revvity, used by five screening programs (13.2%); (4) the NeoBase™ 2 non-derivatized MSMS kit from Revvity, used by eleven screening programs (28.9%); and (5) the laboratory-developed tests, used by ten screening programs (26.3%). Unfortunately, the used assay of one program (Poland) could not be clarified from the questionnaire and additional emails.

#### 3.2.5. Cut-Off per Analytical Assay

In addition to calculating the pooled median cut-off for SUAC and Tyr, we also calculated the median cut-offs per analytical assay. Results can be found in Figure 2 and Figure 3. The highest median cut-off was seen for the NBS programs using LDTs (median: 2.63 µmol/L [SUAC]/210 µmol/L [Tyr]). The SUAC cut-off was significantly higher than the median cut-off from the other analytical assays. 

#### 3.2.6. Incidence, True Positives, False Positives, and False Negatives

The incidence of TT1, according to our results, ranged from 1/13,636 in Canada, Ontario, to 1/750,000 in Poland. The PPV from the different screening programs using SUAC as a biomarker ranged from 0.9% (Hannover, Germany) to 100% (several programs). The PPV from the different screening programs using Tyr as a biomarker ranged from 1.2% (Slovakia) to 66.7% (North Macedonia and Poland) when using Tyr only and from 60% to 100% when using SUAC as a second tier. Combined, all programs using SUAC as primary screening screened 22,084,410 children. Combined, they reported 304 TP results and 811 FP results, yielding a combined PPV for SUAC TT1 screening of 27.3%. Combined, all programs screening with Tyr as the primary marker and SUAC as the secondary marker screened 5,273,431 children. Combined, they reported ten TP results and one FP result, yielding a combined PPV for Tyr + SUAC screening of 90.1%. Lastly, combined, all programs screening with Tyr solely screened 443,051 children. Combined, they reported 1 TP result and 85 FP results, yielding a combined PPV of 1.2%. For the programs using SUAC as a biomarker, only the Netherlands reported a single false-negative result (NPV 99.9%) when having a cut-off value of 1.20 µmol/L. Three FN results were reported for NBS programs using Tyr as the only marker.

#### 3.2.7. Analytical Assays/Methods in Relation to FP Screening Results

To see if there was a relationship between the FP screening results and the used analytical assays, we estimated a combined incidence-based FP-rate for each aforementioned category of analytical assays. This was achieved by dividing the reported total number of screened children per assay by the number of reported FP results per assay and for the different groups of analytical assays (SUAC 1st, Tyr 1st + SUAC 2nd, and Tyr 1st) (Figure 2 and Figure 3). The highest FP-rate (1:6108) was calculated for the derivatized MSMS kit from Chromsystems, Masschrom in programs using Tyr as sole screening marker, followed by a FP-rate of 1:13,651 for the LDT’s in programs using SUAC as primary marker, a FP-rate of 1:14,285 the LDT’s in programs using Tyr as primary marker, a FP-rate of 1:16,434 for the non-derivatized MSMS kit from Chromsystems, Masschrom in programs using SUAC as sole screening marker, a FP-rate of 1:19,643 for the derivatized MSMS kit from Chromsystems, Masschrom, in programs using SUAC as sole marker), a FP-rate of 1:21,919 for the non-derivatized MSMS kit from Chromsystems, Masschrom in programs using Tyr as sole screening marker, a FP-rate of 1:83,310 for the NeoBase™ Non-derivatized MSMS kit from Revvity in programs using SUAC as sole marker, and a FP-rate of 1:127,531 for the NeoBase™ 2 non-derivatized MSMS kit from Revvity in programs using SUAC as sole marker. There did not seem to be a correlation between the time of sampling for NBS and the FP-rate, yet this was difficult to assess as sampling times were given in overlapping ranges of hours after birth.

#### 3.2.8. Significant Changes in the Screening Programs for TT1

Representatives of the screening programs were asked to elaborate on the changes that were made to their screening program for TT1 over the years. The most mentioned changes were (1) change in cut-off (mentioned seven times); (2) change from Tyr to SUAC as screening marker (mentioned six times); and (3) change in analytical assay or mass spectrometer (mentioned six times).

#### 3.2.9. Repeat Samples

Participants from the different screening programs were also asked if they request repeat samples and, if so, for which reason(s), if they participate in external quality control assessments, and if they are informed on the definite diagnosis after a positive screening result from their program. Twenty out of 38 (52.6%) programs request repeat samples, mostly after abnormal screening results. Eight (8/38 = 21.0%) do not request repeat samples. Seven (7/38 = 18.4%) only request repeat samples on some occasions (e.g., system controls, prematurity, abnormal results). All programs participate in external quality assessments for TT1, and from the 35 programs with results to this question, 33 (33/35 = 94.3%) are informed on the diagnosis in case of an abnormal result, as opposed to two programs (3/35 = 5.7%): the Netherlands and the USA, Kansas, who are not directly informed on the diagnosis. 

#### 3.2.10. CDC Data

**Figure 4:**
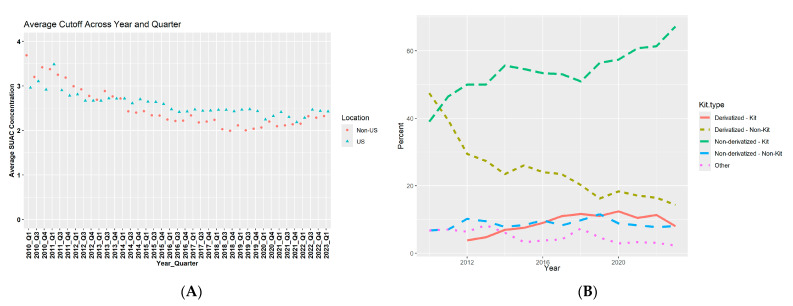
An overview of SUAC cut-offs and used analytical methods for SUAC quantification in US and non-US laboratories internationally (CDC data). (**A**) shows the evolution of average SUAC cut-offs from 2010-2023 per quarter (3 months), differentiating between US and non-US laboratories. (**B**) shows the (evolution of) used analytical methods from 2010–2023.

In addition to our survey, the CDC supplied blinded data from participants on their proficiency testing program. We received the average SUAC cut-off from both US and international non-US labs (*n* = 160) from 2010 until 2023, and the trends in used analytical methods for SUAC quantification over the years. We observed a downward trend over the years in cut-off, which is somewhat more pronounced in the non-US laboratories compared to US laboratories (Figure 4A). Also, laboratories switched over the years from derivatized LDTs to analyze SUAC concentrations to a (non-derivatized) manufacturer-developed kit for analysis (Figure 4B).

## 4. Discussion

TT1 NBS programs vary worldwide in terms of analytical methods, biochemical markers, and cut-offs. Nonetheless, a clear overview of current screening practices for TT1 NBS is lacking [12]. With this study, we aimed to summarize procedures and performances from different NBS programs for TT1 worldwide. These results may provide insights into the cause of, as well as optimization options for, the low PPV observed in the Dutch NBS program for TT1. Moreover, the results from this study will hopefully also inspire other programs worldwide to critically assess their NBS for TT1.

Before discussing the results of our study, some limitations should be mentioned. First of all, we tried to give a complete overview of all countries/regions currently screening for TT1. However, our awareness of who is screening for TT1 in the NBS is dependent on contacts with the authors and the ISNS and the reported research on the NBS for TT1. Especially in Asia and South America, this overview might not be complete. Therefore, we might have unwillingly missed some countries or regions that do screen for TT1 without being included in this study. Moreover, despite our efforts, four of the contacted representatives, including those in the Quebec region, did not respond to our survey, and results from Germany were difficult to collect since they employ regional NBS, of which some regional representatives did not respond to our request to fill in the survey. This resulted in non-response bias, which may affect the validity of this study, as the results might not be generalizable to the entire NBS programs for TT1 worldwide. Our results confirmed the widespread differences in TT1 NBS across the different screening programs worldwide. The first aspect that differed majorly between programs was the cut-offs for Tyr and SUAC. Differences in cut-offs may have several reasons, among which are the use of different analytical assays (LDTs or commercially available kits), the use of different sample preparation protocols (derivatization or not) and mass spectrometers (vendor and/or model), whether or not laboratories account for metabolite recovery or background noise, the use of different internal standards, and differences in calibration procedures [18,19,20,21]. All these analytical factors, as well as the regional population demographics such as prevalence and genetic variants, influence the laboratory’s metabolite cut-off for a specific disease [22].

Large variations in SUAC recoveries, which were previously shown by De Jesus et al. and Adam et al. [23,24], may explain the differences in SUAC cut-offs seen in this study. These variations in recovery can be caused by differences in analytical methods, including derivatized versus non-derivatized methods [12,23,24]. For derivatization, analytes are chemically modified, often using butyl esterification techniques, to enhance chromatographic separation, volatility, stability, and recovery [25]. Higher quantitative SUAC results are measured with derivatized methods, accompanied by higher SUAC cut-offs, while non-derivatized methods measure lower quantitative SUAC results and are usually accompanied with lower SUAC cut-offs to avoid wrongful classification [23,24]. The lower cut-offs in non-derivatized methods were also seen in the results from our study (although the mean differences between the derivatized and non-derivatized kits from Chromsystems were only small: 0.10 µmol/L). Because derivatization is labor-intensive, NBS programs have been switching to non-derivatized methods that allow more high-throughput workflows. Both the gradual adaptation to non-derivatized methods and the simultaneous decrease in SUAC cut-offs were shown in this study.

In addition to the analytical methods, our results did suggest that the use of LDTs is associated with higher cut-offs. LDTs are clinical laboratory tests that are developed, validated, and used within one clinical laboratory but may also include modifications to commercially available assays [26]. They are often developed as a response to unmet analytical or clinical needs and are often helping with obtaining a commercially available test. One advantage of LDTs is that they are flexible to adaptations of different substances, which can be especially worthwhile in rare diseases when testing volumes might be low and availability of commercial kits is limited, often due to the lack of financial rewards for the manufacturers due to small markets [26,27]. Nonetheless, there are concerns from, among others, the Food and Drug Administration (FDA) that low-quality LDTs with poor accuracy and precision of the desired analytical can give rise to FP and FN results, which was shown in their 2015 report, highlighting 20 case studies of undesirable outcomes from LDTs [28]. To regulate the production and use of in vitro diagnostics, including LDTs, and thereby ensure patient safety and public health, the European Commission introduced the ‘In Vitro medical Devices Regulation’ (IVDR) in 2017 (implemented May 2022) [29]. In order for LDTs to be allowed for use, the IVDR requires laboratories, among other things, to specify that specific patient needs cannot be met (with a certain level of performance) with commercially available assays or kits. Similar to the European IVDR act, the USA also recently issued a final rule to enhance safety and effectiveness for LDTs [30]. Although the Medical Device Amendments were already installed in the US by the FDA in 1976, rules have generally not been enforced for LDTs. The final rule (issued May 2024) clarifies that IVDs, including the LDTs, are considered devices under the Federal Food, Drug, and Cosmetic Act and installs increased oversight for LDTs over a phased approach (starting from May 2025) [30]. The requirement from the IVDR is causing a shift of use from LDTs to commercially available kits, which was also seen from the CDC data. A similar shift may be expected for US laboratories when oversight for LDTs is increased over the coming years.

The second main finding of this study was that, surprisingly, the PPVs of many programs using SUAC were much lower than had been reported in the review from Stinton et al., in which the PPV ranged from 66.7% to 100% [12]. In our study, 15 out of 36 (41.7%) NBS programs using SUAC had a PPV < 60%, and the combined PPV of all programs screening with SUAC as the primary marker was 27.3%. Our results also showed very low PPVs for programs using Tyr. Both FP and FN results are known to occur more often in NBS programs using Tyr, and they are one of the main reasons why many programs switched to TT1 screening using SUAC [9,31,32,33].

Low PPVs may occur if the metabolite cut-off for the screening marker is too low. Therefore, one would expect that the largest number of FP test results from our study would be found in the screening programs with the lowest cut-off for SUAC or Tyr. Nonetheless, such results were not consistently found in our study. Instead, high FP-rates seemed to be more related to the used analytical assay, with the highest FP-rates (for SA) seen among the programs using the non-derivatized MSMS kit from Chromsystems, Masschrom, and by those using LDTs. FP SUAC results, in these cases, may be due to poor recovery, calibration or calculation errors, or impurities or contamination of the mass spectrometer causing high background noise [13]. Regarding the non-derivatized method from Chromsystems, the CDC NSQAP quality control program indeed showed lower recoveries for non-derivatized SUAC methods, causing a possible bias and loss of sensitivity in the lower concentrations range. Regarding the LDTs, high FP results, as said before, may also be due to poor-quality LDTs with low precision for measurements near the cut-off. However, it should be noted that the low PPV for LDTs in our study was overall, yet two programs using LDTs, namely Israel and Canada, British Columbia, had high PPVs (90.1% and 75.0%, respectively). This may highlight the large differences in quality among LDTs.

The higher number of FP results in some screening programs could also be due to finding other diseases (both SUAC and Tyr) or elevated metabolite concentrations due to transient physiological states (Tyr) causing metabolite concentrations above the cut-off. With regard to other diseases, SUAC concentrations may be elevated in newborns due to a maleylacetoacetate isomerase (MAAI) deficiency, a seemingly non-pathological condition [34,35,36]. In the Netherlands, MAAI deficiency is a common cause of FP results [35]. It could be that other countries or regions with high FP results, such as Germany, the Philippines, and Ontario, Canada, also have a high prevalence of MAAI deficiency. As SUAC in MAAI deficiency is often only mildly elevated and significantly lower than in TT1 patients [34,35], this theory might be most plausible for Germany, which uses lower SUAC cut-offs (around 1 µmol/L) rather than the Philippines and Canada, Ontario (both using 5.0 µmol/L). Given that MAAI deficiency is assumed to be a benign condition (Yang 2017, Graham 2024) [34], NBS programs with a high FP-rate could consider increasing their cut-offs for SUAC while having to be aware of the risk of FN results in single TT1 patients with unusually low SUAC in NBS [14].

With regard to physiological states, in benign transient tyrosinemia, Tyr is temporarily elevated in newborns due to a combination of immaturity of the enzyme 4-hydroxyphenylpyruvate dioxygenase (4-HPPD) and a relative ascorbic acid deficiency [37]. Transient tyrosinemia is dependent on gestational age and the sampling time after birth. The influences of sampling time on the FP-rate were difficult to assess in our study, as sampling times were given in overlapping time ranges, yet there seemed to be no clear correlation. Nonetheless, it could be that the PPV of Tyr screening improves when correcting for gestational age, which should be further investigated. Tyr concentrations may also reach the cut-off, for example, in tyrosinemia type II and III, and in other both genetic and non-genetic liver diseases. Finding a disease like tyrosinemia type II could be considered a desired unintended finding.

The data presented in our study may suggest that the large heterogeneity of the screening practices and test performances of the different NBS programs for TT1 worldwide has led to inconsistencies in detecting TT1 worldwide [38]. This highlights opportunities to improve the NBS TT1 programs, which can be achieved in several ways.

In aim of improving TT1 NBS, programs might try to enhance their analytical methods, for example, by harmonization [39]. Harmonization potentially corrects for methodological differences in inter-laboratory variations in NBS results [40]. Earlier, Pickens et al. already showed that harmonization of NBS results helps unify disease cut-offs [41]. Common cut-offs allow for homogeneous interpretation of test results and common action guidelines across the different NBS programs [42,43]. Generally, it is considered that there is no clear genotype/phenotype correlation in TT1. Nonetheless, recently reported milder cases of TT1 might suggest some genotype/phenotype relation [14,44,45]. A possible concern would thus be that the common cut-offs as proposed by the ‘Collaborative Laboratory Integrated Reports’ (CLIR—https://clir.mayo.edu, accessed on 10 June 2024) would not suffice for patients with these rare genetic variants.

Additionally, our results showed that on average, LDTs had significantly higher cut-offs than other assays, which suggests a bias in the measured SUAC concentrations. If properly corrected for, this does not necessarily have to result in lower PPVs of the programs (shown by the results from Israel, with a SUAC cut-off of 7.0 µmol/L and a PPV of 90.1%). However, our study showed that programs using LDTs in general also had relatively higher FP-rates (and lower PPVs), with the exception of Canada, British Columbia, and Israel. This might be a reason for some programs to consider changing to a commercial method. Alternatively, programs and laboratories should work together to improve LDTs or available kits and harmonize outcomes. It has been demonstrated previously that particularly harmonization of the calibration standards improves the high inter-laboratory variability of DBS SUAC methods [41].

Programs might also try to adapt their cut-offs, especially for SUAC. In an effort to lower the number of FP screening results, programs could consider increasing their SUAC cut-off. Nonetheless, this should be performed with caution to prevent an increase in FN results. In the Dutch NBS program, one TT1 patient with a historical FN SUAC result presented with HCC in 2020 (SUAC 1.08 µmol/L, cut-off 1.20 µmol/L, Revvity ND-Neobase assay) [14]. This case could argue against increasing the cut-off, as from a clinical perspective, FP results are inevitably less harmful than FN results [12]. This case also shows that TT1 patients may present with lower-than-expected SUAC concentrations. The cut-off in the Netherlands at the time of screening was somewhat higher than the current cut-off of 3/5 programs using the Neobase assay from Revvity (Figure 2). Nonetheless, two other programs using this assay currently have a higher cut-off and may therefore even be at more risk for an FN screening result. For many programs, a reduction in the SUAC cut-off could therefore be considered.

Available data of biomarkers and demographics could also be used to apply an unbiased statistical approach to find the most optimal biomarker combination to screen for TT1 at a population base [46]. Online resources such as Productivity Tools, which are provided via CLIR, could help identify such optimal biomarkers and help set cut-offs for these screening markers [40,47]. CLIR is a web-based interactive database of NBS results from multiple screening programs (https://clir.mayo.edu). The data in CLIR include cumulative reference intervals for biomarkers, including SUAC and Tyr, and ratios of a variety of biomarkers for several diseases, including TT1. It also contains an integrated score based on the degree of overlap between reference ranges and condition-specific disease ranges, adjusted for birth weight, age, sex, and gestational age [40,47]. Based on these data, CLIR could help determine the action threshold based on worldwide combined reference ranges, as well as the optimal (combination of) screening biomarkers. Moreover, CLIR also provides the possibility to perform a disease risk assessment based on day-to-day metabolite values for patients individually. This could increase the specificity of newborn screening by eliminating benign transient metabolite elevations.

The effectiveness of changing to more optimal screening markers is already shown in previous research that highlights the lowering of FP as well as FN results in the NBS programs for TT1 of many programs due to the change in Tyr to SUAC as a screening marker [12,22]. However, because the PPV in this study seemed higher among the programs screening with Tyr as the primary marker and SUAC as the second tier, it might be suggested that TT1 screening with Tyr + SUAC (second tier) is superior to screening with SUAC as the primary screening marker. Nonetheless, in the past, it has been proven that screening for TT1 with Tyr might result not only in more FP results but also increase the chance of missing TT1 patients, due to the possibility of normal or only moderately increased Tyr concentrations in TT1 patients (lack of specificity for Tyr as a biomarker for TT1), which can also be seen in the informative biomarker plots from CLIR that show a large overlap in Tyr concentrations between healthy individuals and TT1 patients [31,48]. Adding SUAC as a second tier is likely to eliminate many FP results; however, the chance for FN results remains, as these children will not proceed to have SUAC measured. This might be confirmed in our results by the fact that Poland, who screens with Tyr + SUAC (second tier), reported two FN results.

Nonetheless, FP results remain to occur with SUAC as markers as well. Therefore, we might have to look for other informative biomarkers or TT1, such as maleic acid for MAAI deficiency [35]. Other markers that, according to CLIR, might be distinctive between TT1 patients and healthy newborns and could lower FP referrals when included in the screening protocols should be considered as well. Moreover, programs might consider implementing genetic *FAH* testing as a secondary or tertiary tier in NBS. This was already achieved in the Norwegian program [49,50].

Lastly, feedback on the eventual diagnosis (TT1 or not) is important for screening programs and laboratories in evaluating their cut-offs. Fortunately, nearly all screening programs received feedback after the child’s diagnosis. In the Netherlands, a special registry called Neorah is used to register screening results and diagnoses of referred newborns and missed patients. With the participation in the screening program, parents agree on the registry, but they are informed on the opt-out procedure at the moment their child’s data are registered in Neorah.

## 5. Conclusions

TT1 NBS programs vary worldwide in terms of analytical methods, biochemical markers and cut-offs giving heterogeneous and large differences in results. Therefore, it is necessary to harmonize the TT1 NBS programs both in relation to the used method/marker and the cut-off value. There is room for improvement through method standardization, cut-off adaptation and integration of new biomarkers. Further enhancement is likely to be achieved by the application of post-analytical tools.

## Figures and Tables

**Table 1:** Demographics of tyrosinemia type 1 newborn screening per screening program.

Country	Sampling Time After Birth (h)	Collection Paper	Start Year	Marker	Cut-Off (µmol/L)	Total Screened	TP	Incidence (Study Period)	FP	FP-Rate (Incidence)	Reason FP	FN	PPV (%)	NPV (%)	Repeat Samples (Y/N/S **)	Remarks/Changes to Screening Program
**Derivatized MSMS kit, Chromsystems, Masschrom**																
Belgium, Wallonia	48–96	Whatman 903	1980s	Tyr + SUAC	216 0.50	±1,000,000	1	Estimated	0	0	NA	0	100%	100%	Y	TLC ≤ 2007 LC-MS/MS ≥ 2007
Estonia	48–72 (max 120)	Whatman 903	2014	SUAC + Tyr	1.86 319	±120,000	0	-	2	1/60,000	Unexplained	0	0%	100%	N	
Germany, Leipzig *	36–72	Revvity 226	2018	SUAC		104,779 *	0 *	-	31 *	1/3379		0 *	0%	100%	-	
Germany, Weiden	36–72	Revvity 226	2018	SUAC	1.50	166,801	1 (2018–2020)	1/166,801	2 (2018–2020)	1/83,400	NA	0	33.3%	100%	Y	Recall rate (0.2%)
Slovakia		Whatman 903	2015	Tyr	250	399,213	1	1/399,213	83	1/4810	Galactosemia, Prematurity, transient Tyr	0	1.2%	100%	Y	
Spain, Eastern Andalusia	36–48	Whatman 903	2010	SUAC	1.80	433,416	1	1/433,416	7	1/61,917	-MAAI deficiency -Transitory benign Tyr	0	12.5%	100%	Y	Tyr 2010–2014 (250 µmol/L) SUAC > 2014
**Non-derivatized MSMS kit, Chromsystems**																
Austria	36–72	Whatman 903	2002	SUAC	2.00	312,000	2	1/156,000	0	0	NA	unk	100%	-	Y	Tyr 2002–2019 (200 µmol/L) SUAC > 2019
Germany, Dresden *	36–72	Revvity 226	2018	SUAC		44,325 *	0 *	-	2 *	1/22,163		0 *	0%	100%	Y	
Germany, Heidelberg	36–72	Eastern Business Forms, Greenville, USA	2018	SUAC	1.10	702,221	3	1/234,073	63	1/11,146	Unexplained	0	4.5%	100%	Y	
Germany, Saxony anhalt	36–72	Revvity 226	2018	SUAC	1.08	78,000	1	1/78,000	6	1/13,000	Mildly elevated SUAC	0	14.3%	100%	N	
North Macedonia	-	Whatman 903	2014	Tyr	350	43,838	0	-	2	1/21,919	Unexplained	0	60.0%	100%	S	
**Laboratory-developed test (LDT)**																
Canada, British Columbia	24–48	Whatman 903	2010	SUAC	7.00	447,648	6	1/74,608	2	1/223,824	Unexplained	0	75.0%	100%	S	Calibration correcting for low recovery of SUAC included
Canada, Ontario, Ottawa	-	Whatman 903	2006	SUAC + Tyr	5.00 600	2,400,000	176	1/13,636	168	1/14,285	Tyr-3, Cobalamin B, Liver disease, MAAI	0	51.2%	100%	S	
Germany, Hannover	36–72	Revvity 226	2008	SUAC	1.50	1,098,934	4	1/274,734	342	1/3212	Unexplained	0	1.1%	100%	Y	-Change in cut-off: 3.5 µmol/L (derivatized) to 1.5 µmol/L (non-derivatized) -Modified non-derivatized MSMS-kit, Chromsystems, Masschrom
Germany, Munich *	36–72	Revvity 226	2018	SUAC		489,766 *	4 *	1/122,441 *	73 *	1/6709		0 *	5.2%	100%	-	
Hungary	48–72	Whatman 903	2007/2008	Tyr + SUAC	120 2.63	708,419	unknown	-	occurred (n) unknown	-	Liver failure or prematurity	0	-	100%	Y	-Change from non-derivitized to derivitized method with corresponding change in SUAC (2022) -Change MS: API4000QTRAP toAPI4500QTRAP -Derivatized LDT
Israel	36–48	Whatman 903	2008	SUAC	7.00	2,500,000	10	1/250,000	1	1/2,500,000	Unexplained	0	90.1%	100%	N	Non-derivatized LDT
Italy, Tuscany region	48–72	Whatman 903	2002	SUAC	2.00 (SUAC)	SUAC:510,000 Tyr:133,600	4	1/127,500	0 (since SUAC)	0	NA	1 (2006-Tyr)/0-SUAC	100%	100%	Y	-Till 2 µmol/L SUAC mainly background noise. -Change Tyr → SUAC (2007)
New Zealand	24–48 (min 24)	PerkinElmer 226	2006/2022	SUAC	5.50 (2022)/2.50 from august 2023	27,949 (2022)/total 102,597	1	1/102,597	0	0	NA	0	100%	100%	Y	2006–2017(stop): Tyr as marker, with two FN results. Restart 2022. August 2023 change LC/MS-MS instrument
Portugal	48–72 (min 36–max 7 days)	Revvity 226	2006	Tyr + SUAC	210 4.00	1,501,212	6	1/250,202	0	0	NA	0	100%	100%	N	Change Tyr cut-off: 250→210 µmol/L
Spain, Galicia	24–48	Whatman 903	2000	SUAC, SUAC/Tyr, SUAC/Phe, SUAC/Met	1.00 (sometimes 1.50)	450,256	3	1/150,085	0	0	NA	1 (using Tyr)	100% (SUAC)	100% (SUAC)	Y	Change Tyr → SUAC (2008)
**NeoBase™ 2 Non-derivatized MSMS kit, Revvity**																
Belgium, Flanders	72–96	Revvity 226	2022	Tyr + SUAC	175 0.59	63,800	1	1/63,800	0	0	NA	unk	100%	-	Y	-
Denmark	48–72	Revvity 226	2009	SUAC + Tyr	1.60 150	840,000	5	1/168,000	0	0	NA	0	100%	100%	N	
Finland	36–120 (most between 36 and 72)	Revvity 226	2015	SUAC	0.70	400,000	6	1/67,000	0	0	NA	0	100%	100%	N	-Change Neobase → Neobase 2 (2019) + change cut-off 1.34 → 0.7 µmol/L -Change from Waters Xevo TQD (MS) to Waters Xevo TQ-S micro (2023)
Germany, Hamburg	36–72	Revvity 226	2018	SUAC	1.60	242,681	2	1/121,340	1	1/242,681		0	66.7%	100%	Y	
The Netherlands	72–168	Whatman 903	2007	SUAC	0.60	2,358,809	27	1/87,363	35	1/67,395	-Contamination of MS results analytical border -low CUT-OFF VALUE	1	43.4%	99,9%	N	-Water Xevo TQD (MS) -Tyr 2007 (500 µmol/L) Change Tyr → SUAC (2008) -SUAC: 1.5 µmol/L (2008), 1.2 µmol/L (2009), change to Neobase2 (2018, 0.9 µmol/L), 0.6 µmol/L (2019)
Norway	48–72	Whatman 903	2012	SUAC	2.00	700,000	10	1/70,000	4	1/175,000	Reported prior to FAH sequencing results available	0	71.4%	100%	N	*FAH* sequencing as second tier on same DBS → reporting of (FP) result before *FAH* screening
Singapore	24–72	Whatman 903	2006	Tyr + SUAC	150 1.00	>500,000 (total) 147.000 since Neobase 2 (2019)	0	-	0	0	NA	0	-	-	S	-Change Cambridge Isotope derivatized to Neobase 2 (2019) + change cut-off SUAC 5.0 to 1.0 µmol/L -Change AbSciex API 3200 → Osight 210MD
Slovenia	48–72	Whatman 903	2018	SUAC, SUAC/Tyr, SUAC/Phe, SUAC/Met	0.30	74,200	0	-	4	1/18,550	Intensive care admission (other disease)	0	0%	100%	Y	-
Spain, Madrid region	48–72	Whatman 903	2011	SUAC	1.00	702,356	4	1/175,589	8	1/87,794	Unexplained	0	33.3%	100%	S	Change Neobase → Neobase 2 (2018)
Sweden	48–72	Revvity 226	2010	SUAC	2.00	1,376,015	18	1/76,445	1	1/1,376,015	Unexplained	0	94.7%	100%	N	-Waters XevoTQD (MS) -Change Neobase → Neobase 2 (2020)
**NeoBase™ Non-derivatized MSMS kit, Revvity**																
Canada, Alberta	24–72 *** up to 24 months	Whatman 903	2019	SUAC	1.50	178,369	1	1/178,369	0	0	NA	0	100%	100%	S	-
Germany, Berlin *	36–72	Revvity 226	2018	SUAC		178,203 *	3 *	1/59,401 *	9 *	1/19,800	-	0 *	27.3%	100%	Y	
Germany, Greifswald *	36–72	Revvity 226	2018	SUAC	0.90										Y	
Germany, Hessen	36–72	Whatman 903	2020	SUAC	1.00	150,000	0	-	0	0	NA	0	-	100%	Y	-
Philippines	24–48	Whatman 903	2014	SUAC	5.00	4,408,744	10	1/440,874	50 (Mindanao region)	1/88,174	Low cut-off 1.5 µmol/L	unk	16.7%	-	Y	Change cut-off: 1.5 → 5.0 µmol/L -FP only known for Mindanao region
USA, Kansas	<24	Whatman 903	<2016	SUAC	0.96	±280,000 (since 2016)	0	-	0	0	NA	0	-	100%	S	Change in cut-off from 0.70 to 0.96 µmol/L
**Other/not disclosed**																
Germany (whole)	36–72	Lab dependent	2018	SUAC	Lab dependent (11 labs)	±3,800,000	20	1/190,000 2020 (1/110,449) *	521	1/7293	Unexplained	unk	3.7%	-	Lab dep.	Lab dependent method: For regional specifics, see above
Poland	-	Whatman 903	2018	Tyr + SUAC	400	>1,500,000	2	1/750,000	1	1/1,500,000	Unexplained	2	66.7%	99.9%	Y	SUAC second tier if Tyr > 200 µmol/L 2 FN results <2017, using Tyr as marker

* Up to 2020, from the German NBS reports (DGNS, there was no personal response to the survey. ** Sometimes can be for several reasons, e.g., only if results are abnormal (British Colombia, North Macedonia, Poland, Madrid (Spain), and Kansas (USA)). Ontario (Canada) only asks repeat samples in prematurity, and Sweden, as well as Finland, only asks repeat samples as system control samples. *** In Alberta, Canada, the upper age limit for screening is 24 months, allowing the inclusion of infants from international adoptions and infants from new immigrants and refugee families. Abbreviations: false positives; FP, false negatives; FN, true positives; TP, liquid chromatography mass spectrometry; LC-MS/MS, mass spectrometry; MS, negative predictive value; NPV, positive predictive value; PPV, succinylacetone (SUAC); TLC, thin-layer chromatography; Tyr, tyrosine; MAAI, maleylacetoacetate isomerase deficiency; unk, unknown.

## Data Availability

The data presented in this study are available upon reasonable request from the corresponding authors.

## Supplementary Materials

The following supporting information can be downloaded at: https://www.mdpi.com/article/10.3390/ijns10040082/s1. File S1: overview of survey questions; File S2: CHERRIES checklist.

## Appendix A

1. Conchita G. Abarquez, Newborn Screening Center Mindanao, Southern Philippines Medical Center, Davao City, Philippines; conchabarquez@yahoo.com
2. Violeta Anastasovska, University Pediatric Clinic, Department for neonatal screening, 1000 Skopje, North Macedonia; violeta_anastasovska@yahoo.com
3. Shlomo Almashanu, Newborn Screening Laboratories, Tel-HaShomer, 52621 Ramat Gan, Israel; shlomo.almashanu@moh.gov.il
4. François Boemer, Biochemical Genetics Laboratory, CHU Liège, University of Liège, 4000 Liège, Belgium; f.boemer@chuliege.be
5. Inken Brockow, Screening Center, Bavarian Health, and Food Safety Authority (LGL), Veterinaerstrasse 2, 85764, Oberschleissheim, Germany; Inken.brockow@lgl.bayern.de
6. Ana Cambra Conejero, Laboratorio de Cribado Neonatal de la Comunidad de Madrid. Servicio de Bioquímica Clínica. Hospital General Universitario Gregorio Marañón, Madrid, España; ana.cambra@salud.madrid.org
7. Uta Ceglarek, Institute of Laboratory Medicine, Clinical Chemistry and Molecular Diagnostics, University Hospital Leipzig, Germany; uta.ceglarek@medizin.uni-leipzig.de
8. Carla Cuthbert, Newborn Screening and Molecular Biology Branch, Centers for Disease Control and Prevention; ijz6@cdc.gov
9. Francois Eyskens, Kon. Mathilde Moeder- en Kindcentrum, University Hospital of Antwerp, Antwerp, Belgium; francois.eyskens@uza.be
10. Ralph Fingerhut, SYNLAB MVZ Weiden GmbH, Zur Kesselschmiede 4, 92637 Weiden, Germany; ralph.fingerhut@synlab.com
11. Ewa Głąb-Jabłońska, Institute of Mother and Child, 01-211 Warsaw, Poland; ewa@rglab.pl
12. Urh Groselj, University of Ljubljana, Faculty of Medicine; UMC Ljubljana—University Children’s Hospital, Ljubljana, Slovenia; urh.groselj@kclj.si
13. Mark de Hora, Newborn Metabolic Screening Unit, Auckland City Hospital Auckland New Zealand; mdehora@adhb.govt.nz
14. Friederike Hörster, Heidelberg University, Medical Faculty of Heidelberg, Department of Pediatrics 1, Division of Pediatric Neurology and Metabolic Medicine; Newborn screening; Heidelberg, Germany; friederike.hoerster@med.uni-heidelberg.de
15. Gwendolyn Gramer, Simona Murko, Newborn Screening and Metabolic Laboratory, University Children’s Hospital, University Medical Center Eppendorf Hamburg Germany; s.murko@uke.de, g.gramer@uke.de
16. David Hougaard, Staten Serum Institute, 2300 Copenhagen, Denmark; dh@ssi.dk
17. Nils Janzen, Screening Laboratory Hannover, Box 91 10 09, 30430 Hannover, Germany; n.janzen@metabscreen.de
18. Mária Knapková, Newborn Screening Centre, Banska Bystrica 97401, Slovakia; maria.knapkova@dfnbb.sk
19. Riikka Kurkijärvi, Finnish National Newborn Screening Centre (Saske), Tyks Laboratories Department of Genomics, Turku University Hospital, 20521 Turku, Finland; riikka.kurkijarvi@tyks.fi
20. Giancarlo la Marca, Newborn Screening, Clinical Chemistry and Pharmacology Lab, Meyer Children’s Hospital IRCCS, Department of Experimental and Clinical Biomedical Sciences, Univeristy of Florence 50139 Florence, Italy; g.lamarca@meyer.it
21. Nathalie Lepage, Department of Pathology and Laboratory Medicine, University of Ottawa, Ottawa, Canada; nlepage@cheo.on.ca
22. James S. Lim, KK Women’s and Children’s Hospital, Singapore; james.lim.sc@kkh.com.sg
23. Martin Lindner, Division of Metabolic Diseases, University Children’s Hospital Frankfurt, Frankfurt, Germany; martin.lindner@kgu.de
24. Allan M. Lund, Department of Clinical Medicine, Faculty of Health and Clinical Sciences, Copenhagen University, Copenhagen, Denmark; allan.lund@regionh.dk
25. Gwendolyn McKee, Tennessee Department of Health, Division of Laboratory Services; gwendolyn.mckee@tn.gov
26. Cristóbal Colón, Laboratorio de Metabolopatias. Hospital Clínico Universitario. Santiago de Compostela. España; cristobal.colon.mejeras@sergas.es
27. Ruth Mikelsaar, Karit Reinson, Tartu University Hospital, Genetics and Personalized Medicine Clinic, 50406, Tartu, Estonia: University of Tartu, Faculty of Medicine, Institute of Clinical Medicine, 50406, Tartu, Estonia; karit.reinson@kliinikum.ee; ruth.mikelsaar@ut.ee
28. Michelle Mills, Kansas Health and Environmental Laboratories Newborn Screening Program; michelle.j.mills@ks.gov
29. Barbka Repic Lampret, Ljubljana University Medical Centre, University Children’s Hospital, 1000 Ljubljana, Slovenia; barbka.repic@kclj.si
30. Sabine Rönicke, Institut für Klinische Chemie und Pathobiochemie, University clinic, Magdeburg, Germany; sabine.roenicke@med.ovgu.de
31. Graham Sinclair, Dept. of Pathology and Laboratory Medicine, BC Children’s Hospital. Vancouver, Canada; gsinclair@cw.bc.ca
32. Iveta Sosova, Alberta Newborn Screening and Biochemical Genetics Laboratories, Edmonton, AB, Canada; iveta.sosova@albertaprecisionlabs.ca
33. Asbjørg Stray-Pedersen, Norwegian National Unit for Newborn Screening, Oslo 0424, Norway; astraype@ous-hf.no
34. Ildiko Szatmari, Semmelweis University Department of Pediatrics, Budapest, Hungary; szatmari.ildiko@semmelweis.hu
35. Laura Vilarinho, National Institute of Health Dr. Ricardo Jorge (INSA), Porto; laura.vilarinho@insa.min-saude.pt
36. Theresa Winter, Institute for Clinical Chemistry and Laboratory Medicine, University Medicine Greifswald, Greifswald, Germany; theresa.winter@med.uni-greifswald.de
37. Raquel Yahyaoui, Laboratory of Inherited Metabolic Diseases and Newborn Screening. Malaga Regional University Hospital. Institute of Biochemical Research in Malaga and Platform of Nanomedicine (IBIMA platform BIONAND), 29011, Malaga, Spain; raquelyahyaoui@gmail.com
38. Rolf Zetterström, Centre for Inherited Metabolic Diseases, Karolinska University Hospital and Department of Molecular Medicine and Surgery, Karolinska Institute, SE-17 76 Stockholm, Sweden; rolf.zetterstrom@sll.se
39. Maximilian Zeyda, Department of Pediatrics and Adolescent Medicine, 1090 Vienna, Austria; maximilian.zeyda@meduniwien.ac.at
