# Correction: Kuypers et al. Evaluation of Neonatal Screening Programs for Tyrosinemia Type 1 Worldwide. *Int. J. Neonatal Screen.* 2024, *10*, 82

**DOI:** 10.3390/ijns11030072

**Published:** 2025-09-01

**Authors:** Allysa M. Kuypers, Marelle J. Bouva, J. Gerard Loeber, Anita Boelen, Eugenie Dekkers, Konstantinos Petritis, C. Austin Pickens, The ISNS Representatives, Francjan J. van Spronsen, M. Rebecca Heiner-Fokkema

**Affiliations:** 1 Section of Metabolic Diseases, Beatrix Children’s Hospital, University Medical Center Groningen, University of Groningen, 9700 RB Groningen, The Netherlands; a.m.dijkstra@umcg.nl (A.M.K.);; 2 Centre for Health Protection, National Institute for Public Health and the Environment (RIVM), 3720 BA Bilthoven, The Netherlands; marelle.bouva@rivm.nl; 3 International Society for Neonatal Screening (ISNS) Office, 3721 CK Bilthoven, The Netherlands; 4 Endocrine Laboratory, Department of Clinical Chemistry, Amsterdam Gastroenterology, Endocrinology & Metabolism, Amsterdam UMC, University of Amsterdam, 1105 AZ Amsterdam, The Netherlands; 5 Centre for Population Research, National Institute for Public Health and the Environment (RIVM), 3720 BA Bilthoven, The Netherlands; 6 Newborn Screening and Molecular Biology Branch, Division of Laboratory Sciences, National Center for Environmental Health, Centers for Disease Control and Prevention, Atlanta, GA 30341, USA; 7 Laboratory of Metabolic Diseases, Department of Laboratory Medicine, University Medical Center Groningen, University of Groningen, P.O. Box 30 001, 9700 RB Groningen, The Netherlands

The authors wish to make the following correction to their paper published in the *International Journal of Neonatal Screening* [1].

1. In the Abstract, overall positive predictive values for SUAC should be 25.9% instead of 27.3%;

2. In the Introduction, the sentence “Moreover, since the start of SUAC NBS for TT1 in the Netherlands, 57% of positive screening results have proven to be FP results, and in 2020, a false-negative (FN) TT1 patient emerged” should be updated to “Moreover, since the start of SUAC NBS for TT1 in the Netherlands, 82% of positive screening results have proven to be FP results, and in 2020, a false-negative (FN) TT1 patient emerged”;

3. In Table 1, the TP of the Netherlands should be 11 instead of 27, the incidence (study period) should be 1/214,437 instead of 1/87,363, the FP results should be 49 instead of 35, the FP rate (incidence) should be 1/48,139 instead of 1/67,395, and the PPV (%) should be 18.3% instead of 43.4%;

4. The authors would like to replace Figure 2 with the following figure; only the FP rate of the Netherlands was changed from 1/67,395 to 1/48,139;




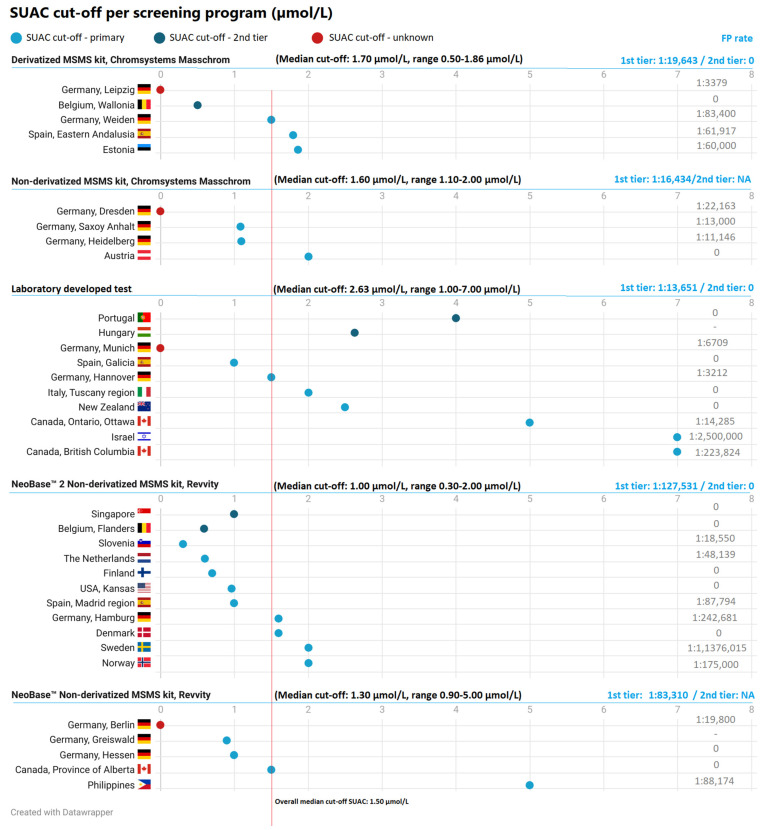




5. In Section 3.2.6, the sentence “Combined, they reported 304 TP results and 811 FP results, yielding a combined PPV for SUAC TT1 screening of 27.3%. Combined, all programs screening with Tyr as the primary marker and SUAC as the secondary marker screened 5,273,431 children” should be updated to “Combined, they reported 288 TP results and 825 FP results, yielding a combined PPV for SUAC TT1 screening of 25.9%. Combined, all programs screening with Tyr as the primary marker and SUAC as the secondary marker screened 5,273,431 children”;

6. In Section 3.2.7, the sentence “a FP-rate of 1:83,310 for the NeoBase™ Non-derivatized MSMS kit from Revvity in programs using SUAC as sole marker, and a FP-rate of 1:127,531 for the NeoBase™ 2 non-derivatized MSMS kit from Revvity in programs using SUAC as sole marker” should be updated to “a FP-rate of 1:83,310 for the NeoBase™ Non-derivatized MSMS kit from Revvity in programs using SUAC as sole marker, and a FP-rate of 1:103,058 for the NeoBase™ 2 non-derivatized MSMS kit from Revvity in programs using SUAC as sole marker”;

7. In the Discussion, the sentence “In our study, 15 out of 36 (41.7%) NBS programs using SUAC had a PPV < 60%, and the combined PPV of all programs screening with SUAC as the primary marker was 27.3%” should be updated to “In our study, 15 out of 36 (41.7%) NBS programs using SUAC had a PPV < 60%, and the combined PPV of all programs screening with SUAC as the primary marker was 25.9%”;

8. In the backmatter section, the Institutional Review Board Statement should be updated to “Institutional Review Board Statement: The Institutional Review Board Statement was waived because this study did not include clinical research with individual human subjects as meant by the Dutch Medical Research Act involving human subjects”. The Informed Consent Statement should be updated to “Informed Consent Statement: The Informed Consent Statement was waived because this study did not include clinical research with individual human subjects as meant by the Dutch Medical Research Act involving human subjects”.

We would like to apologize for any inconvenience caused to the readers by these changes. The changes do not affect the scientific results. This correction was approved by the Academic Editor. The original publication has also been updated.

## Appendix A

1. Conchita G. Abarquez, Newborn Screening Center Mindanao, Southern Philippines Medical Center, Davao City, Philippines; conchabarquez@yahoo.com
2. Violeta Anastasovska, University Pediatric Clinic, Department for neonatal screening, 1000 Skopje, North Macedonia; violeta_anastasovska@yahoo.com
3. Shlomo Almashanu, Newborn Screening Laboratories, Tel-HaShomer, 52621 Ramat Gan, Israel; shlomo.almashanu@moh.gov.il
4. François Boemer, Biochemical Genetics Laboratory, CHU Liège, University of Liège, 4000 Liège, Belgium; f.boemer@chuliege.be
5. Inken Brockow, Screening Center, Bavarian Health, and Food Safety Authority (LGL), Veterinaerstrasse 2, 85764, Oberschleissheim, Germany; Inken.brockow@lgl.bayern.de
6. Ana Cambra Conejero, Laboratorio de Cribado Neonatal de la Comunidad de Madrid. Servicio de Bioquímica Clínica. Hospital General Universitario Gregorio Marañón, Madrid, España; ana.cambra@salud.madrid.org
7. Uta Ceglarek, Institute of Laboratory Medicine, Clinical Chemistry and Molecular Diagnostics, University Hospital Leipzig, Germany; uta.ceglarek@medizin.uni-leipzig.de
8. Carla Cuthbert, Newborn Screening and Molecular Biology Branch, Centers for Disease Control and Prevention; ijz6@cdc.gov
9. Francois Eyskens, Kon. Mathilde Moeder- en Kindcentrum, University Hospital of Antwerp, Antwerp, Belgium; francois.eyskens@uza.be
10. Ralph Fingerhut, SYNLAB MVZ Weiden GmbH, Zur Kesselschmiede 4, 92637 Weiden, Germany; ralph.fingerhut@synlab.com
11. Ewa Głąb-Jabłońska, Institute of Mother and Child, 01-211 Warsaw, Poland; ewa@rglab.pl
12. Urh Groselj, University of Ljubljana, Faculty of Medicine; UMC Ljubljana—University Children’s Hospital, Ljubljana, Slovenia; urh.groselj@kclj.si
13. Mark de Hora, Newborn Metabolic Screening Unit, Auckland City Hospital Auckland New Zealand; mdehora@adhb.govt.nz
14. Friederike Hörster, Heidelberg University, Medical Faculty of Heidelberg, Department of Pediatrics 1, Division of Pediatric Neurology and Metabolic Medicine; Newborn screening; Heidelberg, Germany; friederike.hoerster@med.uni-heidelberg.de
15. Gwendolyn Gramer, Simona Murko, Newborn Screening and Metabolic Laboratory, University Children’s Hospital, University Medical Center Eppendorf Hamburg Germany; s.murko@uke.de, g.gramer@uke.de
16. David Hougaard, Staten Serum Institute, 2300 Copenhagen, Denmark; dh@ssi.dk
17. Nils Janzen, Screening Laboratory Hannover, Box 91 10 09, 30430 Hannover, Germany; n.janzen@metabscreen.de
18. Mária Knapková, Newborn Screening Centre, Banska Bystrica 97401, Slovakia; maria.knapkova@dfnbb.sk
19. Riikka Kurkijärvi, Finnish National Newborn Screening Centre (Saske), Tyks Laboratories Department of Genomics, Turku University Hospital, 20521 Turku, Finland; riikka.kurkijarvi@tyks.fi
20. Giancarlo la Marca, Newborn Screening, Clinical Chemistry and Pharmacology Lab, Meyer Children’s Hospital IRCCS, Department of Experimental and Clinical Biomedical Sciences, Univeristy of Florence 50139 Florence, Italy; g.lamarca@meyer.it
21. Nathalie Lepage, Department of Pathology and Laboratory Medicine, University of Ottawa, Ottawa, Canada; nlepage@cheo.on.ca
22. James S. Lim, KK Women’s and Children’s Hospital, Singapore; james.lim.sc@kkh.com.sg
23. Martin Lindner, Division of Metabolic Diseases, University Children’s Hospital Frankfurt, Frankfurt, Germany; martin.lindner@kgu.de
24. Allan M. Lund, Department of Clinical Medicine, Faculty of Health and Clinical Sciences, Copenhagen University, Copenhagen, Denmark; allan.lund@regionh.dk
25. Gwendolyn McKee, Tennessee Department of Health, Division of Laboratory Services; gwendolyn.mckee@tn.gov
26. Cristóbal Colón, Laboratorio de Metabolopatias. Hospital Clínico Universitario. Santiago de Compostela. España; cristobal.colon.mejeras@sergas.es
27. Ruth Mikelsaar, Karit Reinson, Tartu University Hospital, Genetics and Personalized Medicine Clinic, 50406, Tartu, Estonia: University of Tartu, Faculty of Medicine, Institute of Clinical Medicine, 50406, Tartu, Estonia; karit.reinson@kliinikum.ee; ruth.mikelsaar@ut.ee
28. Michelle Mills, Kansas Health and Environmental Laboratories Newborn Screening Program; michelle.j.mills@ks.gov
29. Barbka Repic Lampret, Ljubljana University Medical Centre, University Children’s Hospital, 1000 Ljubljana, Slovenia; barbka.repic@kclj.si
30. Sabine Rönicke, Institut für Klinische Chemie und Pathobiochemie, University clinic, Magdeburg, Germany; sabine.roenicke@med.ovgu.de
31. Graham Sinclair, Dept. of Pathology and Laboratory Medicine, BC Children’s Hospital. Vancouver, Canada; gsinclair@cw.bc.ca
32. Iveta Sosova, Alberta Newborn Screening and Biochemical Genetics Laboratories, Edmonton, AB, Canada; iveta.sosova@albertaprecisionlabs.ca
33. Asbjørg Stray-Pedersen, Norwegian National Unit for Newborn Screening, Oslo 0424, Norway; astraype@ous-hf.no
34. Ildiko Szatmari, Semmelweis University Department of Pediatrics, Budapest, Hungary; szatmari.ildiko@semmelweis.hu
35. Laura Vilarinho, National Institute of Health Dr. Ricardo Jorge (INSA), Porto; laura.vilarinho@insa.min-saude.pt
36. Theresa Winter, Institute for Clinical Chemistry and Laboratory Medicine, University Medicine Greifswald, Greifswald, Germany; theresa.winter@med.uni-greifswald.de
37. Raquel Yahyaoui, Laboratory of Inherited Metabolic Diseases and Newborn Screening. Malaga Regional University Hospital. Institute of Biochemical Research in Malaga and Platform of Nanomedicine (IBIMA platform BIONAND), 29011, Malaga, Spain; raquelyahyaoui@gmail.com
38. Rolf Zetterström, Centre for Inherited Metabolic Diseases, Karolinska University Hospital and Department of Molecular Medicine and Surgery, Karolinska Institute, SE-17 76 Stockholm, Sweden; rolf.zetterstrom@sll.se
39. Maximilian Zeyda, Department of Pediatrics and Adolescent Medicine, 1090 Vienna, Austria; maximilian.zeyda@meduniwien.ac.at
