# Supplementary material for: Evaluation of Neonatal Screening Programs for Tyrosinemia Type 1 Worldwide
Source: Int J Neonatal Screen. 2024 Dec 16;10(4):82. doi: 10.3390/ijns10040082 (PMC11677071; doi:10.3390/ijns10040082)
Supplement: Supplementary file 1 [file IJNS-10-00082-s001.zip › File S1 Survey questions TT1 survey.pdf]

The following questions will be regarding the TT1 screening and laboratory analyses in your country/center.

*

1. This survey is distributed among countries that have implemented screening for TT1. For which country/center are you filling in this questionnaire?

*

2. In what year was TT1 screening implemented in your country/center?

*

3. When are NBS samples taken in your country? (sampling time: please indicate after ... hours or days)

4. Which marker is used for TT1 screening in your country/center?

- Succinylacetone (SA)
- Tyrosine (TYR)
- Other, for example a combination of SA/TYR or a different marker:

*

5. What assay (method) is used for TT1 screening (Manufacturer/Kit/Batch etc.)?

6. What type of collection paper is used for the neonatal screening programme in your country/center?

- Whatman 903
- PerkinElmer 226
- Other:

7. Are metabolite concentrations in DBS measured in µM(µmol/L) serum or µM(µmol/L) blood?

- Blood
- Serum
- If applicable Other:

8. What cut-off value is used to obtain a positive screening result? (please include measuring unit behind your answer)

9. Do you request repeat DBS samples for TT1 screening?

- Yes
- No
- Under certain circumstances:

10. Does your lab participate in an external quality assessment programme concerning TT1?

- Yes
- No

11. Were there any changes in kits/methods/manufacturers or cut-off values since the implementation of TT1 screening in your country/center? Please choose the answers to such changes:

- Important changes in cutoff limits:
- Prominent analytical developments including changes in equipment:
- Number of screening laboratories in your country:
- Other:
- No changes

12. How many children in total were screened for TT1 in your country thus far? (Also indicate if this is not known)

13. How many children in total were screened positive for TT1 thus far? (Also indicate if this is not known)

*

14. How many children in total had a **false-positive** screening result for TT1 thus far? (Also indicate if this is not known)

*

Which were the reasons for a false positive TT1 test result? (multiple answers possible)

- Other diagnosis, namely:
- Analytical, namely:
- other:
- Do not know
- Not applicable

*

15. How many children in total had a **false-negative** screening result for TT1 thus far? (Also indicate if this is not known)

16. After a positive TT1 neonatal screening result, children are referred to a hospital for further investigation. Are you (as coordinating member of the neonatal screening/ head of screening laboratory of other member involved in the neonatal screening) informed about the eventual diagnosis?

- Yes
- No
